# Untreated Psychiatric and Substance Use Disorders Among Caregivers With Children Reported to Child Protective Services

**DOI:** 10.1001/jamahealthforum.2024.0637

**Published:** 2024-04-19

**Authors:** Tami L. Mark, Melissa Dolan, Benjamin Allaire, William Parish, Claire Strack, Diana Poehler, Emily Madden, Valeria Butler

**Affiliations:** 1 RTI International, Washington, DC; 2 Office of the Assistant Secretary for Planning and Evaluation, Department of Health and Human Services, Washington, DC; 3 Office of Planning, Research and Evaluation, Administration for Children and Families, Department of Health and Human Services, Washington, DC

## Abstract

**Importance:**

Mental and substance use disorders can interfere with parents’ ability to care for their children and are associated with a greater likelihood of child protective services involvement to address child maltreatment. Parent engagement in psychiatric and substance use disorder treatment can prevent child maltreatment and family separations.

**Objective:**

To determine whether caregivers with psychiatric or substance use disorders whose children were referred to child protective services received Medicaid-funded psychiatric or substance use disorder treatment.

**Design, Setting, and Participants:**

Caregivers listed on child welfare records were linked with their Medicaid records using 2017 to 2020 Medicaid and child welfare data from Florida and Kentucky. Medicaid claims were analyzed to determine if caregivers had a psychiatric or substance use disorder diagnosis and whether those caregivers received counseling or medications. The analysis was conducted in 2023.

**Exposure:**

Diagnosis of a psychiatric or substance use disorder in 2020.

**Main Outcome and Measure:**

Receipt of psychiatric or substance use disorder counseling or medications.

**Results:**

Of the 58 551 caregivers, 65% were aged between 26 and 40 years; 69% were female and 31% were male. Overall, 78% identified as White, 20% identified as Black/African American, and less than 1% identified as American Indian/Alaska Native, Asian, or Native Hawaiian/Other Pacific Islander. In 2020, 59% of caregivers with Medicaid and children referred to child protective services had a mental health or substance use disorder diagnosis, compared with 33% of age- and sex-matched Medicaid beneficiaries without children referred to child protective services (*P* < .001). Among caregivers with a psychiatric disorder, 38% received counseling and 67% received psychiatric medication. Among those with a substance use disorder, 40% received counseling and 38% received a substance use disorder medication.

**Conclusions and Relevance:**

In this case-control study, despite Medicaid coverage of an array of effective behavioral health treatments, large portions of caregivers with Medicaid coverage, who need treatment and whose children were referred to child protective services, were not receiving treatment. Medicaid and child welfare agencies should make a greater effort to connect caregivers to behavioral health services.

## Key Points

**Question:**

To what extent do caregivers with Medicaid coverage whose children were referred to child protective services as at risk of maltreatment receive mental health or substance use disorder treatment?

**Findings:**

In this case-control study, less than 50% of caregivers with Medicaid coverage with mental health or substance use disorders whose children were referred to child protective services received counseling or substance use disorder medications.

**Meaning:**

Medicaid and child welfare programs should enhance caregiver receipt of mental health and substance use disorder treatment to improve parenting skills and prevent child maltreatment and family separations.

## Teaser

This case-control study examines the extent to which caregivers with Medicaid coverage whose children were referred to child protective services as at risk of maltreatment receive mental health or substance use disorder treatment.

## Introduction

Child welfare agencies aim to protect children from the profound socioemotional, behavioral, cognitive, and health effects resulting from family maltreatment and neglect.^1,2^ In 2021, there were 3 987 000 referrals to child protective services (CPS) alleging maltreatment involving 7 176 600 children, and 206 000 children were removed from their homes because of maltreatment.^3^

Congress and state governments are directing more resources to try to prevent childhood maltreatment and neglect without separating children from their families. Prevention programs aim to alleviate the risk factors for child maltreatment and neglect such as caregiver mental health (MH) and substance use disorders (SUD); economic stress such as housing, food, and employment insecurity; and lack of parenting skills.^4^ For example, the Family First Prevention Services Act (FFPSA) of 2018 allows states to use Title IV-E child welfare funds to pay for evidence-based MH and SUD treatment and in-home parent skill-based programs.^5^ The federal government is also encouraging greater coordination between child welfare and other social service programs, such as Medicaid.^6^ Medicaid covers 36% of all children in the US, 16% of all women aged 18 to 64 years, and most families with incomes below the poverty line.^7^ Medicaid benefits include a range of evidence-based MH and SUD therapies that have been shown to improve parental MH, SUD, and parenting skills, and in turn, positively affect children’s outcomes.^8^

This study used new, unique data—the Child and Caregiver Outcomes Using Linked Data (CCOULD)—to evaluate the receipt of Medicaid-funded MH and SUD treatment among caregivers with MH/SUD conditions with CPS involvement. To our knowledge, CCOULD is the first dataset to link caregivers involved in the child welfare system with their Medicaid records.

## Methods

### Data Sources and Description

The CCOULD contains child welfare records linked with Medicaid enrollment and claims data in Kentucky and Florida for January 2017 through June 2021 in Florida, and January 2017 through January 2020 in Kentucky. A full description of CCOULD and how the Medicaid and child welfare child and caregiver data were linked is available in a published government report.^9^ The data include Medicaid enrollment and claims files that capture child and caregivers’ health care diagnoses and health care service use as well as child welfare files that include maltreatment reports such as the outcome of the CPS investigation (eg, whether it was substantiated or not), types of services provided to families, and whether children were removed from their home. Caregivers of children were identified on the child welfare reports and linked to the caregivers’ Medicaid records using the caregivers’ social security numbers. The institutional review board of RTI International determined that the project was exempt from review because all data were deidentified.

### Medicaid Non–Child Welfare Sample

The dataset also included a 10% random sample of adult and child Medicaid beneficiaries who did not have records in the child welfare system. These data allowed for comparisons between adults with and without children referred to CPS. Whether the adults in the non–child welfare Medicaid sample have children not referred to CPS or do not have children at all, cannot be determined because Medicaid does not include a variable that identifies whether the Medicaid beneficiary is a parent.

### Statistical Analysis

We identified caregivers with an MH, SUD, opioid use disorder (OUD), or alcohol use disorder (AUD) using primary or secondary diagnosis codes on the Medicaid claims. To compare the diagnosis rates among caregivers with children referred to CPS, we matched each caregiver to an adult in the random Medicaid sample file using 1:1 direct matching on age, sex, and enrollment year. We calculated the standardized differences in demographics to ensure equivalent groups. Receipt of MH counseling was identified using procedure codes on the Medicaid data (see Supplement 1 for a list of codes). Receipt of MH, SUD, or OUD medications was identified using Medicaid prescription drug claims or medical claims. We tested for differences in mean rates among these groups using a 2-sample *t* test for proportions and corrected for multiple comparisons using a Bonferroni adjustment. We present the results for 2020 herein. Results for 2018 and 2019 are included in Supplement 1.

## Results

Of the 58 551 caregivers, 65% were aged between 26 and 40 years; 69% were female and 31% were male. Overall, less than 1% identified as American Indian or Alaska Native, Asian, Native Hawaiian or Other Pacific Islander, 20% identified as Black or African American, and 78% identified as White (Table 1). The percentage of Hispanic or Latino caregivers in the dataset was 5%, although 17% of the sample had missing ethnicity information.

**Table 1.** Characteristics of 58 551 Caregivers With Children Investigated by Child Protective Services and Enrolled in Medicaid and 58 551 Age/Sex-Matched Adults in Medicaid, 2020

Characteristic	No. (%)	
	Caregivers with children involved in child welfare	Age- and sex-matched adults without children involved with child welfare
Kentucky	33 786 (58)	33 786 (58)
Florida	24 765 (42)	24 765 (42)
Age group, y		
18-25	8342 (14)	8342 (14)
26-40	38 124 (65)	38 124 (65)
41-55	10 959 (19)	10 959 (19)
56-64	863 (1)	863 (1)
≥65	1 (0)	1 (0)
Missing	262 (0)	262 (0)
Sex		
Male	18 061 (31)	18 061 (31)
Female	40 489 (69)	40 489 (69)
Race		
American Indian or Alaska Native	122 (0)	95 (0)
Asian	164 (0)^a^	677 (1)
Black or African American	11 740 (20)^a^	11 025 (19)
Native Hawaiian/Other Pacific Islander	70 (0)^a^	1633 (3)
White	45 499 (78)^a^	31 959 (55)
Missing	956 (2)^a^	13 162 (22)
Ethnicity		
Hispanic or Latino	3199 (5)^a^	7186 (12)
Not Hispanic or Latino	45 583 (78)^a^	46 745 (80)
Missing	9769 (17)^a^	4620 (8)
MH or SUD diagnoses		
MH or SUD	34 696 (59)^a^	19 256 (33)
MH	26 453 (45)^a^	16 520 (28)
SUD	24 463 (42)^a^	7043 (12)
Opioid use disorder	17 312 (30)^a^	4646 (8)
Alcohol use disorder	10 624 (18)^a^	3035 (5)

Abbreviations: MH, mental health; SUD, substance use disorder.

^a^ *P* < .001.

The prevalence of MH, SUD, OUD, and AUD was much higher among Medicaid enrollees with children referred to CPS than age- and sex-matched Medicaid enrollees without children referred to CPS (Table 1). The prevalence of an MH diagnosis was 1.6 times greater in the CPS population than the matched sample (45% compared with 28%); SUD was 3.5 times greater (42% compared with 12%); OUD was 3.8 greater (30% compared with 8%), and AUD was 3.6 times greater (18% compared with 5%).

Most caregivers did not receive MH or SUD counseling or SUD medications (Table 2). Among caregivers with an MH, SUD, or OUD diagnosis, 38%, 40%, and 44% received any counseling, respectively. Only 38% and 52% of caregivers with an SUD or OUD diagnosis received an SUD or OUD medication, respectively. Receipt of MH medications was more common with 67% of those with an MH diagnosis receiving MH medication.

**Table 2.** Receipt of Medicaid-Funded Behavioral Health Services Among Caregivers With Child Protective Services Involvement, With Psychiatric, Substance Use, or Opioid Use Disorders (n = 58 551), 2020

Variable	%		
	With MH diagnosis	With SUD diagnosis	With OUD diagnosis
Any MH/SUD counseling	38	40	44
Any MH medications	67	52	53
Any SUD medications	25	38	53
Any OUD medications	24	38	52

Abbreviations: MH, mental health; OUD, opioid use disorder; SUD, substance use disorder.

## Discussion

This study found a large, unmet need for MH/SUD counseling and MH/SUD medications among caregivers with children involved in child welfare. Addressing this unmet need would benefit caregivers and their children, and prevent child maltreatment and family separations.^8^ An important step in closing this gap is greater coordination between child welfare and Medicaid agencies, such as through integrating child welfare and Medicaid data systems so that child welfare can ensure that caregivers are receiving Medicaid services as needed. Furthermore, interventions are needed to address the barriers that caregivers face in obtaining MH/SUD treatment, such as stigma, inconvenience, financial hardship, and fear of losing parental rights.^10^ Moreover, because caregivers involved in the child welfare system typically have multiple complex needs, programs should deliver comprehensive integrated care that addresses caregivers’ parenting skills as well as medical, financial, and other social needs rather than just focusing on treating their SUD or MH.^11,12^

### Limitations

Although the analyses used a unique dataset, they have some limitations. One limitation is that Medicaid claims data do not capture MH and SUD services that caregivers might receive that are funded by other sources, such as through federal block grants, state budgets, or private payers. A second limitation is that claims data only capture MH/SUD that were diagnosed and recorded by a clinician. Claims data miss undiagnosed MH/SUD.

## Conclusions

Child welfare agencies are tasked with protecting children from maltreatment while trying to prevent family separations. This case-control study suggests that many Medicaid-covered caregivers needing MH and SUD treatments, whose children were referred to CPS, were not receiving treatment despite Medicaid covering effective options. In the future, Medicaid and child welfare agencies should make a greater effort to connect caregivers to MH and SUD services.

## Notes

Supplement 1. **eAppendix.** List of Codes Used to Identify Medications, Counseling, and a Mental Health, Substance Use Disorder, or Opioid Use Disorder Diagnosis **eTable 1.** Characteristics of Caregivers With Children Investigated by Child Protective Services and Enrolled in Medicaid (n = 64 239) and Age/Gender Matched Adults in Medicaid (n = 64 239), 2018 **eTable 2.** Characteristics of Caregivers With Children Investigated by Child Protective Services and Enrolled in Medicaid (n = 60 054) and Age/Gender Matched Adults in Medicaid (n = 60 054), 2019 **eTable 3.** Receipt of Medicaid-Funded Behavioral Health Services Among Caregivers With Child Protective Services Involvement, With Psychiatric, Substance Use, or Opioid Use Disorders (n = 64 239), 2018 **eTable 4.** Receipt of Medicaid-Funded Behavioral Health Services Among Caregivers With Child Protective Services Involvement, With Psychiatric, Substance Use, or Opioid Use Disorders (n = 60 054), 2019

Supplement 2.
Data Sharing Statement
