# Supplementary material for: Untreated Psychiatric and Substance Use Disorders Among Caregivers With Children Reported to Child Protective Services
Source: JAMA Health Forum. 2024 Apr 19;5(4):e240637. doi: 10.1001/jamahealthforum.2024.0637 (PMC11065155; doi:10.1001/jamahealthforum.2024.0637)
Supplement: Supplement 1. — eAppendix. List of Codes Used to Identify Medications, Counseling, and a Mental Health, Substance Use Disorder, or Opioid Use Disorder Diagnosis eTable 1. Characteristics of Caregivers With Children Investigated by Child Protective Services and Enrolled in Medicaid (n = 64 239) and Age/Gender Matched Adults in Medicaid (n = 64 239), 2018 eTable 2. Characteristics of Caregivers With Children Investigated by Child Protective Services and Enrolled in Medicaid (n = 60 054) and Age/Gender Matched Adults in Medicaid (n = 60 054), 2019 eTable 3. Receipt of Medicaid-Funded Behavioral Health Services Among Caregivers With Child Protective Services Involvement, With Psychiatric, Substance Use, or Opioid Use Disorders (n = 64 239), 2018 eTable 4. Receipt of Medicaid-Funded Behavioral Health Services Among Caregivers With Child Protective Services Involvement, With Psychiatric, Substance Use, or Opioid Use Disorders (n = 60 054), 2019 [file jamahealthforum-e240637-s001.pdf]

Supplemental Online Content

Mark TL, Dolan M, Allaire B, et al. Untreated psychiatric and substance use disorders among caregivers with children reported to Child Protective Services. *JAMA Health Forum*. 2024;5(4):e240637. doi:10.1001/jamahealthforum.2024.0637

eAppendix. List of Codes Used to Identify Medications, Counseling, and a Mental Health, Substance Use Disorder, or Opioid Use Disorder Diagnosis

eTable 1. Characteristics of Caregivers With Children Investigated by Child Protective Services and Enrolled in Medicaid (n = 64 239) and Age/Gender Matched Adults in Medicaid (n = 64 239), 2018

eTable 2. Characteristics of Caregivers With Children Investigated by Child Protective Services and Enrolled in Medicaid (n = 60 054) and Age/Gender Matched Adults in Medicaid (n = 60 054), 2019

eTable 3. Receipt of Medicaid-Funded Behavioral Health Services Among Caregivers With Child Protective Services Involvement, With Psychiatric, Substance Use, or Opioid Use Disorders (n = 64 239), 2018

eTable 4. Receipt of Medicaid-Funded Behavioral Health Services Among Caregivers With Child Protective Services Involvement, With Psychiatric, Substance Use, or Opioid Use Disorders (n = 60 054), 2019

This supplemental material has been provided by the authors to give readers additional information about their work.

© 2024 Mark TL et al. *JAMA Health Forum.*

eAppendix. List of Codes Used to Identify Medications, Counseling, and a Mental Health, Substance Use Disorder, or Opioid Use Disorder Diagnosis

Mental Health Medications

Alprazolam
Alprazolam C-IV
Amitriptyline Hydrochloride
Amoxapine
Amphetamine
Amphetamine Aspartate
Amphetamine Sulfate
Apraclonidine
Aripiprazole
Aripiprazole Lauroxil
Armodafinil
Asenapine
Asenapine Maleate
Atomoxetine
Brexanolone
Brexpiprazole
Bupropion Hydrobromide
Bupropion Hydrochloride
Buspirone Hydrochloride
Carbamazepine
Cariprazine
Chlordiazepoxide And Amitriptyline Hydrochloride
Chlordiazepoxide Hcl And Clidinium Bromide
Chlordiazepoxide Hydrochloride
Chlordiazepoxide Hydrochloride And Clidinium Bromide
Chlorpromazine
Citalopram
Citalopram Hydrobromide
Clomipramine Hydrochloride
Clonazepam
Clonidine
Clonidine Hydrochloride
Clorazepate Dipotassium
Clozapine
Desipramine Hydrochloride
Desvenlafaxine

© 2024 Mark TL et al. *JAMA Health Forum.*

Desvenlafaxine Succinate
Dexmethylphenidate Hydrochloride
Dextroamphetamine
Dextroamphetamine Saccharate, Amphetamine Aspartate Monohydrate, Dextroamphetamine Sulfate And Amphetamine Sulfate
Dextroamphetamine Saccharate, Amphetamine Aspartate Monohydrate, Dextroamphetamine Sulfate, Amphetamine Sulfate Er
Dextroamphetamine Saccharate, Amphetamine Aspartate Monohydrate, Dextroamphetamine Sulfate, And Amphetamine Sulfate
Dextroamphetamine Saccharate, Amphetamine Aspartate, Dextroamphetamine Sulfate And Amphetamine Sulfate
Dextroamphetamine Saccharate, Amphetamine Aspartate, Dextroamphetamine Sulfate, And Amphetamine Sulfate
Dextroamphetamine Sulfate
Dextroamphetamine Sulfate, Dextroamphetamine Saccharate, Amphetamine Sulfate And Amphetamine Aspartate
Dextromethorphan Hydrobromide, Bupropion Hydrochloride
Diazepam
Doxepin
Doxepin Hydrochloride
Droperidol
Duloxetine
Duloxetine And Lidocaine
Escitalopram
Escitalopram Oxalate
Esketamine Hydrochloride
Estazolam
Flumazenil
Fluoxetine
Fluphenazine Decanoate
Fluphenazine Hydrochloride
Flurazepam Hydrochloride
Fluvoxamine Maleate
Guanfacine
Haloperidol
Haloperidol Decanoate
Haloperidol Lactate
Hydroxyamphetamine Hydrobromide, Tropicamide
Hydroxyzine
Hydroxyzine Pamoate
Imipramine Hydrochloride
Imipramine Pamoate

© 2024 Mark TL et al. *JAMA Health Forum.*

Isocarboxazid
Iloperidone
Lamotrigine
Levomilnacipran
Lisdexamfetamine Dimesylate
Lithium Carbonate
Lorazepam
Loxapine
Lurasidone Hydrochloride
Maprotiline Hydrochloride
Meprobamate
Methamphetamine Hydrochloride
Methylphenidate
Midazolam
Mirtazapine
Modafinil
Molindone Hydrochloride
Naltrexone Hydrochloride And Bupropion Hydrochloride
Nefazodone Hydrochloride
Nortriptyline
Olanzapine
Olanzapine And Samidorphan L-Malate
Oxazepam
Oxcarbazepine
Paliperidone
Paliperidone Palmitate
Paroxetine
Paroxetine Hydrochloride Hemihydrate
Paroxetine Mesylate
Perphenazine
Phenelzine
Pimavanserin Tartrate
Pimozide
Prochlorperazine
Prochlorperazine Edisylate
Prochlorperazine Maleate
Protriptyline Hydrochloride
Quazepam
Quetiapine
Quetiapine Fumarate
Risperidone
Selegiline

© 2024 Mark TL et al. *JAMA Health Forum.*

Sensor Aripiprazole
Serdexmethylphenidate And Dexmethylphenidate
Sertraline
Sprinkle Duloxetine
Temazepam
Thioridazine
Thiothixene
Tranylcypromine
Tranylcypromine Sulfate
Trazodone Hydrochloride
Triazolam
Trifluoperazine
Trimipramine
Trimipramine Maleate
Venlafaxine
Vilazodone Hydrochloride
Vortioxetine
Ziprasidone
Ziprasidone Mesylate

Opioid Medications

Buprenorphine
Buprenorphine/naloxone
Methadone
Naloxone
Naltrexone

Alcohol Medications

Acamprosate Calcium
Disulfiram
Naltrexone

Diagnoses

Diagnosis	ICD-10 Codes
Mental Health Condition	F0, F2, F3, F4, F5, F6, F7, F8, or F9
Opioid Use Disorder	F11, T40-T40.6

© 2024 Mark TL et al. *JAMA Health Forum.*

Alcohol Use Disorder	F10, G62.1, I42.6, K29.2, K70.0-K70.4, K70.9, T51.0X1A-T51.0X4A, Z71.41, O99.3, O35.4XX0-O35.4XX5, O35.4XX9
Substance Use Disorder	F10 -F16; F18 -F19, G62.1; I42.6; K29.2; K70.0-K70.4; K70.9; T51.0X1A- T51.0X4A; Z71.41; O99.3; O35.4XX0-O35.4XX5; O35.4XX9; T40.0-T40.6; T40.7X1A; T40.8X1A; T40.901A; T40.991A; O99.3; O35.5XX

Counseling Procedures

**Counseling Procedure Codes**	**Code Description**
90804	Individual Psychotherapy, Insight Oriented, Behavior Modifying And/Or Supportive, In An Office Or Outpatient Facility, Approximately 20 To 30 Minutes Face-To-Face With The Patient;
90805	Individual Psychotherapy, Insight Oriented, Behavior Modifying And/Or Supportive, In An Office Or Outpatient Facility, Approximately 20 To 30 Minutes Face-To-Face With The Patient; With Medical Evaluation And Management Services
90806	Individual Psychotherapy, Insight Oriented, Behavior Modifying And/Or Supportive, In An Office Or Outpatient Facility, Approximately 45 To 50 Minutes Face-To-Face With The Patient;
90807	Individual Psychotherapy, Insight Oriented, Behavior Modifying And/Or Supportive, In An Office Or Outpatient Facility, Approximately 45 To 50 Minutes Face-To-Face With The Patient; With Medical Evaluation And Management Services
90808	Individual Psychotherapy, Insight Oriented, Behavior Modifying And/Or Supportive, In An Office Or Outpatient Facility, Approximately 75 To 80 Minutes Face-To-Face With The Patient;
90809	Individual Psychotherapy, Insight Oriented, Behavior Modifying And/Or Supportive, In An Office Or Outpatient Facility, Approximately 75 To 80 Minutes Face-To-Face With The Patient; With Medical Evaluation And Management Services
90810	Individual Psychotherapy, Interactive, Using Play Equipment, Physical Devices, Language Interpreter, Or Other Mechanisms Of Non-Verbal Communication, In An Office Or Outpatient Facility, Approximately 20 To 30 Minutes Face-To-Face With The Patient;
90811	Individual Psychotherapy, Interactive, Using Play Equipment, Physical Devices, Language Interpreter, Or Other Mechanisms Of Non-Verbal Communication, In An Office Or Outpatient Facility, Approximately 20 To 30 Minutes Face-To-Face With The Patient; With Medical Evaluation And Management Services

© 2024 Mark TL et al. *JAMA Health Forum.*

90812	Individual Psychotherapy, Interactive, Using Play Equipment, Physical Devices, Language Interpreter, Or Other Mechanisms Of Non-Verbal Communication, In An Office Or Outpatient Facility, Approximately 45 To 50 Minutes Face-To-Face With The Patient;
90813	Individual Psychotherapy, Interactive, Using Play Equipment, Physical Devices, Language Interpreter, Or Other Mechanisms Of Non-Verbal Communication, In An Office Or Outpatient Facility, Approximately 45 To 50 Minutes Face-To-Face With The Patient; With Medical Evaluation And Management Services
90814	Individual Psychotherapy, Interactive, Using Play Equipment, Physical Devices, Language Interpreter, Or Other Mechanisms Of Non-Verbal Communication, In An Office Or Outpatient Facility, Approximately 75 To 80 Minutes Face-To-Face With The Patient;
90815	Individual Psychotherapy, Interactive, Using Play Equipment, Physical Devices, Language Interpreter, Or Other Mechanisms Of Non-Verbal Communication, In An Office Or Outpatient Facility, Approximately 75 To 80 Minutes Face-To-Face With The Patient; With Medical Evaluation And Management Services
90816	Individual Psychotherapy, Insight Oriented, Behavior Modifying And/Or Supportive, In An Inpatient Hospital, Partial Hospital Or Residential Care Setting, Approximately 20 To 30 Minutes Face-To-Face With The Patient;
90817	Individual Psychotherapy, Insight Oriented, Behavior Modifying And/Or Supportive, In An Inpatient Hospital, Partial Hospital Or Residential Care Setting, Approximately 20 To 30 Minutes Face-To-Face With The Patient; With Medical Evaluation And Management Services
90818	Individual Psychotherapy, Insight Oriented, Behavior Modifying And/Or Supportive, In An Inpatient Hospital, Partial Hospital Or Residential Care Setting, Approximately 45 To 50 Minutes Face-To-Face With The Patient;
90819	Individual Psychotherapy, Insight Oriented, Behavior Modifying And/Or Supportive, In An Inpatient Hospital, Partial Hospital Or Residential Care Setting, Approximately 45 To 50 Minutes Face-To-Face With The Patient; With Medical Evaluation And Management Services
90821	Individual Psychotherapy, Insight Oriented, Behavior Modifying And/Or Supportive, In An Inpatient Hospital, Partial Hospital Or Residential Care Setting, Approximately 75 To 80 Minutes Face-To-Face With The Patient;
90822	Individual Psychotherapy, Insight Oriented, Behavior Modifying And/Or Supportive, In An Inpatient Hospital, Partial Hospital Or Residential Care Setting, Approximately 75 To 80 Minutes Face-To-Face With The Patient; With Medical Evaluation And Management Services
90823	Individual Psychotherapy, Interactive, Using Play Equipment, Physical Devices, Language Interpreter, Or Other Mechanisms Of Non-Verbal Communication, In An Inpatient Hospital, Partial Hospital Or Residential Care Setting, Approximately 20 To 30 Minutes Face-To-Face With The Patient;

© 2024 Mark TL et al. *JAMA Health Forum.*

90824	Individual Psychotherapy, Interactive, Using Play Equipment, Physical Devices, Language Interpreter, Or Other Mechanisms Of Non-Verbal Communication, In An Inpatient Hospital, Partial Hospital Or Residential Care Setting, Approximately 20 To 30 Minutes Face-To-Face With The Patient; With Medical Evaluation And Management Services
90825	Psychiatric Evaluation/Records-Reports Bundled 7/1/96
90826	Individual Psychotherapy, Interactive, Using Play Equipment, Physical Devices, Language Interpreter, Or Other Mechanisms Of Non-Verbal Communication, In An Inpatient Hospital, Partial Hospital Or Residential Care Setting, Approximately 45 To 50 Minutes Face-To-Face With The Patient;
90827	Individual Psychotherapy, Interactive, Using Play Equipment, Physical Devices, Language Interpreter, Or Other Mechanisms Of Non-Verbal Communication, In An Inpatient Hospital, Partial Hospital Or Residential Care Setting, Approximately 45 To 50 Minutes Face-To-Face With The Patient; With Medical Evaluation And Management Services
90828	Individual Psychotherapy, Interactive, Using Play Equipment, Physical Devices, Language Interpreter, Or Other Mechanisms Of Non-Verbal Communication, In An Inpatient Hospital, Partial Hospital Or Residential Care Setting, Approximately 75 To 80 Minutes Face-To-Face With The Patient;
90829	Individual Psychotherapy, Interactive, Using Play Equipment, Physical Devices, Language Interpreter, Or Other Mechanisms Of Non-Verbal Communication, In An Inpatient Hospital, Partial Hospital Or Residential Care Setting, Approximately 75 To 80 Minutes Face-To-Face With The Patient; With Medical Evaluation And Management Services
90832	Psychotherapy, 30 Minutes
90833	Psychotherapy, 30 Minutes
90834	Psychotherapy, 45 Minutes
90836	Psychotherapy, 45 Minutes
90837	Psychotherapy, 60 Minutes
90838	Psychotherapy, 60 Minutes
90839	Psychotherapy For Crisis, First 60 Minutes
90840	Psychotherapy For Crisis; Each Additional 30 Minutes (List Separately In Addition To Code For Primary Service)
90845	Psychoanalysis
90846	Family Psychotherapy, 50 Minutes
90847	Family Psychotherapy Including Patient, 50 Minutes
90849	Multiple-Family Group Psychotherapy
90853	Group Psychotherapy (Other Than Of A Multiple-Family Group)
90857	Interactive Group Psychotherapy
99354	Prolonged Office Or Other Outpatient Service First Hour

© 2024 Mark TL et al. *JAMA Health Forum.*

H2012	Behavioral Health Day Treatment, Per Hour
H2019	Therapeutic Behavioral Services, Per 15 Minutes
H2033	Multisystemic Therapy For Juveniles, Per 15 Minutes
S9480	Intensive Outpatient Psychiatric Services, Per Diem
S9482	Family Stabilization Services, Per 15 Minutes
S9484	Crisis Intervention Mental Health Services, Per Hour

© 2024 Mark TL et al. *JAMA Health Forum.*

eTable 1. Characteristics of Caregivers With Children Investigated by Child Protective Services and Enrolled in Medicaid (n = 64 239) and Age/Gender Matched Adults in Medicaid (n = 64 239), 2018

	Caregivers with children involved in child welfare		Age/gender matched adults w/o children involved with child welfare	
	**Count**	**Percent**	**Count**	**Percent**
**Kentucky**	34,945	54%	34,945	54%
**Florida**	29,294	46%	29,294	46%
**Age groups**				
Age 18-25	13,859	22%	13,859	22%
Age 26-40	40,274	63%	40,274	63%
Age 41-55	8,796	14%	8,796	14%
Age 56-64	645	1%	645	1%
Age 65+	0	0%	0	0%
Missing	665	1%	665	1%
**Gender**				
Male	19,202	30%	19,202	30%
Female	45,036	70%	45,036	70%
**Race**				
American Indian or Alaska Native	146	0%	102	0%
Asian	186	0%	701	1%
Black or African American	13,055	20%	12,394	19%
Native Hawaiian/Other Pacific Islander	68	0%	1,787	3%
White	49,746	77%	34,635	54%
Missing	1,038	2%	14,620	23%
**Ethnicity**				
Hispanic or Latino	3,723	6%	8,477	13%
Not Hispanic or Latino	49,389	77%	51,120	80%
Missing	11,127	17%	4,642	7%
**Any Mental Health (MH) or Substance Use Disorder (SUD) Diagnoses**				
	**Caregivers with children involved in child welfare**		**Age/gender matched adults w/o children involved with child welfare**	
	**Count**	**Percent**	**Count**	**Percent**
MH or SUD	36,164	56%***	19,617	31%
MH	26,866	42%***	16,564	26%
SUD	24,772	39%***	7,066	11%
Opioid Use Disorder	17,898	28%***	4,681	7%
Alcohol Use Disorder	11,798	18%***	3,186	5%

© 2024 Mark TL et al. *JAMA Health Forum.*

NOTES: The standardized differences were less than 0.1 for all matched categories. Race was not matched. Differences in MH and SUD prevalence rates between populations were statistically significant after adjusted for multiple comparisons. *** $p < .001$

© 2024 Mark TL et al. *JAMA Health Forum.*

eTable 2. Characteristics of Caregivers With Children Investigated by Child Protective Services and Enrolled in Medicaid (n = 60 054) and Age/Gender Matched Adults in Medicaid (n = 60 054), 2019

	Caregivers with children involved in child welfare		Age/gender matched adults w/o children involved with child welfare	
	Count	Percent	Count	Percent
**Kentucky**	33,465	56%	33,465	56%
**Florida**	26,589	44%	26,589	44%
**Age groups**				
Age 18-25	10,931	18%	10,931	18%
Age 26-40	38,501	64%	38,501	64%
Age 41-55	9,526	16%	9,526	16%
Age 56-64	713	1%	713	1%
Age 65+	1	0%	1	0%
Missing	382	1%	382	1%
**Gender**				
Male	17,868	30%	17,868	30%
Female	42,184	70%	42,184	70%
**Race**				
American Indian or Alaska Native	136	0%	92	0%
Asian	172	0%	661	1%
Black or African American	12,206	20%	11,502	19%
Native Hawaiian/Other Pacific Islander	68	0%	1,740	3%
White	46,474	77%	32,722	54%
Missing	998	2%	13,337	22%
**Ethnicity**				

© 2024 Mark TL et al. *JAMA Health Forum.*

	Caregivers with children involved in child welfare		Age/gender matched adults w/o children involved with child welfare	
	**Count**	**Percent**	**Count**	**Percent**
Hispanic or Latino	3,382	6%	7,568	13%
Not Hispanic or Latino	46,435	77%	48,135	80%
Missing	10,237	17%	4,351	7%
**Any Mental Health (MH) or Substance Use Disorder (SUD) Diagnoses**				
MH or SUD	35,091	58%***	19,547	33%
MH	26,219	44%***	16,619	28%
SUD	24,632	41%***	7,326	12%
Opioid Use Disorder	17,823	30%***	4,833	8%
Alcohol Use Disorder	11,264	19%***	3,235	5%

NOTES: The standardized differences were less than 0.1 for all matched categories. Race was not matched. Differences in MH and SUD prevalence rates between populations were statistically significant after adjusted for multiple comparisons. *** $p < .001$

© 2024 Mark TL et al. *JAMA Health Forum.*

eTable 3. Receipt of Medicaid-Funded Behavioral Health Services Among Caregivers With Child Protective Services Involvement, With Psychiatric, Substance Use, or Opioid Use Disorders (n = 64 239), 2018

	**With MH diagnosis**	**With SUD diagnosis**	**With OUD diagnosis**
Any MH/SUD counseling	35%	36%	38%
Any MH medications	65%	48%	49%
Any SUD medications	17%	28%	38%
Any OUD medications	17%	28%	38%

© 2024 Mark TL et al. *JAMA Health Forum.*

eTable 4. Receipt of Medicaid-Funded Behavioral Health Services Among Caregivers With Child Protective Services Involvement, With Psychiatric, Substance Use, or Opioid Use Disorders (n = 60 054), 2019

	With MH diagnosis	With SUD diagnosis	With OUD diagnosis
Any MH/SUD counseling	38%	40%	42%
Any MH medications	65%	50%	51%
Any SUD medications	21%	34%	45%
Any OUD medications	21%	33%	45%

© 2024 Mark TL et al. *JAMA Health Forum.*
