# Supplementary material for: Untreated Psychiatric and Substance Use Disorders Among Caregivers With Children Reported to Child Protective Services
Source: JAMA Health Forum. 2024 Apr 19;5(4):e240637. doi: 10.1001/jamahealthforum.2024.0637 (PMC11065155; doi:10.1001/jamahealthforum.2024.0637)
Supplement: Supplement 2. — Data Sharing Statement [file jamahealthforum-e240637-s002.pdf]

Data Sharing Statement

Mark. Untreated Psychiatric and Substance Use Disorders Among Caregivers With Children Reported to Child Protective Services. *JAMA Health Forum*. Published April 19, 2024. doi:10.1001/jamahealthforum.2024.0637

Data
Data available: Yes
Data types: Deidentified participant data, Data dictionary
How to access data: https://www.ndacan.acf.hhs.gov/datasets
When available: With publication

Supporting Documents
Document types: None

Additional Information
Who can access the data: resarchers whos proposed use of the data has been approved
Types of analyses: any analyses that meet the Data Use Agreement
Mechanisms of data availability: See NDACAN website requirements. The data are available for free to researchers as long as they comply wiht the Data Use Agreement
